# Association of Nonsteroidal Anti-inflammatory Drug Use With Survival in Patients With Squamous Cell Carcinoma of the Head and Neck Treated With Chemoradiation Therapy

**DOI:** 10.1001/jamanetworkopen.2020.7199

**Published:** 2020-06-30

**Authors:** Austin J. Iovoli, Gregory M. Hermann, Sung Jun Ma, Alexis J. Platek, Mark K. Farrugia, Edwin Yau, Kimberly E. Wooten, Hassan Arshad, Vishal Gupta, Moni A. Kuriakose, Wesley L. Hicks, Anurag K. Singh

**Affiliations:** 1 Department of Radiation Medicine, Roswell Park Comprehensive Cancer Center, Buffalo, New York; 2 Department of Radiation Medicine, University at Buffalo Jacobs School of Medicine and Biomedical Sciences, The State University of New York, Buffalo; 3 Department of Medical Oncology, Roswell Park Comprehensive Cancer Center, Buffalo, New York; 4 Department of Head and Neck Surgery, Roswell Park Comprehensive Cancer Center, Buffalo, New York

## Abstract

**Importance:**

Nonsteroidal anti-inflammatory drugs (NSAIDs), such as aspirin, are commonly prescribed medications with anti-inflammatory and antiplatelet properties used long term to decrease the risk of cardiovascular events. A recent study showed that aspirin was associated with improved survival in patients with head and neck squamous cell carcinoma (HNSCC) who were treated with surgery.

**Objective:**

To examine whether use of NSAIDs during definitive chemoradiation therapy (CRT) was associated with improved outcomes in patients with HNSCC.

**Design, Setting, and Participants:**

This cohort study analyzed patients with HNSCC who were treated with CRT at a single institution between January 1, 2005, and August 1, 2017. Patient and tumor characteristics included age, race/ethnicity, smoking status, alcohol use, comorbidities (respiratory, cardiovascular, immune, renal, endocrine), disease stage, human papillomavirus status, and treatment duration. Data were analyzed from May 1, 2019, to March 17, 2020.

**Exposures:**

Patients were dichotomized by NSAID use during treatment.

**Main Outcomes and Measures:**

The association of NSAID use with patterns of failure, disease-specific survival (DSS), and overall survival (OS) was examined using multivariate Cox proportional hazard regression models. Survival estimates for OS and DSS were generated using Kaplan-Meier survival curves.

**Results:**

A total of 460 patients (median [interquartile range] age, 60 [53.9-65.6] years; 377 [82.0%] men) were included in the analysis. Among these patients, 201 (43.7%) were taking NSAIDs during treatment. On univariate analysis, NSAID use (hazard ratio [HR], 0.63; 95% CI, 0.43-0.92; *P* = .02) was associated with better OS. On Cox regression analysis, after backward selection adjustment for potentially confounding factors (age, smoking status, primary tumor site, human papillomavirus status, diabetes, stroke, hyperlipidemia), NSAID use remained significantly associated with better OS (HR, 0.59; 95% CI, 0.38-0.90; *P* = .02). NSAID use was associated with significantly better OS at 5 years compared with patients who did not take concurrent NSAIDs (63.6% [56 of 88 patients]; 95% CI, 58%-73% vs 56.1% [83 of 148 patients]; 95% CI, 50%-63%; *P* = .03). NSAID use was not associated with better DSS in univariate (HR, 0.82; 95% CI, 0.48-1.41; *P* = .47) or multivariate (HR, 0.98; 95% CI, 0.57-1.70; *P* = .44) analysis. NSAID use was not associated with better response to treatment (HR, 1.44; 95% CI, 0.91-2.27; *P* = .12) or distant failure (HR, 1.12; 95% CI, 0.68-1.84; *P* = .65). Change in local control with NSAID use was not statistically significant (HR, 0.59; 95% CI, 0.31-1.10; *P* = .10).

**Conclusions and Relevance:**

This cohort study suggests a possible OS advantage for patients taking NSAIDs during chemoradiation for HNSCC. Further studies examining this association are warranted.

## Key Points

**Question:**

Is nonsteroidal anti-inflammatory drug (NSAID) use associated with a survival advantage in patients with head and neck squamous cell carcinoma who are treated with definitive chemoradiation therapy?

**Findings:**

In this cohort study of 460 patients, NSAID use was associated with significantly better overall survival at 5 years compared with no use of concurrent NSAIDs.

**Meaning:**

These findings suggest there may be an overall survival advantage for patients with head and neck squamous cell carcinoma who take NSAIDs during chemoradiation.

## Teaser

This cohort study investigates the association between nonsteroidal anti-inflammatory drug (NSAID) use and survival in patients with head and neck squamous cell carcinoma treated with chemoradiation therapy (CRT) at a single institution between 2005 and 2017.

## Introduction

Head and neck squamous cell carcinoma (HNSCC) is one of the leading causes of cancer death in the United States. There is an increasing incidence of human papillomavirus (HPV)-associated tumors with 53 000 new cases in 2019.^1,2^ HPV-associated tumors more frequently have phosphoinositide 3-kinase (*PI3K*) gene variations independent of tobacco exposure.^3,4^ By exploiting this oncogenic addiction phenotype, aspirin and other nonsteroidal anti-inflammatory drugs (NSAIDs) have been identified as potential chemotherapeutic agents owing to the effect they have on cyclooxygenase inhibition, which is required for prostaglandin synthesis.^5^ Concurrent use of NSAIDs has been shown to be associated with a survival advantage for colorectal cancer and a wide range of cancer histologies.^6^

Whether NSAID use protects against the development of HNSCC remains controversial, as results have been mixed or inconclusive.^7,8,9,10,11^ Recently, however, Hedberg et al found that regular NSAID use at any time improves disease-specific survival (DSS) and overall survival (OS) in patients with *PI3K*-altered HNSCC.^12^ Less explored is the association of NSAID use with definitive chemoradiation therapy (CRT). This cohort study was performed using a large single institution HNSCC database to further examine the survival outcomes associated with NSAID use during CRT.

## Methods

The Roswell Park Comprehensive Cancer Center institutional review board approved this retrospective cohort study of patients with HNSCC diagnosed and treated at a single institution with CRT between January 1, 2005, and August 1, 2017. An approved waiver of consent was obtained from the Roswell Park Comprehensive Cancer Center because the research met the criteria for minimal risk to the study participants. This study did not follow the Strengthening the Reporting of Observational Studies in Epidemiology (STROBE) reporting guideline.

### Eligibility

**Figure 1.**
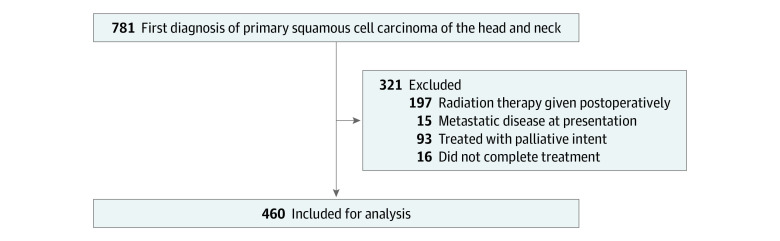
Patient Selection Criteria

To be included in this study, patients had to have the following criteria: (1) a first-time diagnosis of cancer, (2) an invasive squamous cell carcinoma limited to the head and neck, (3) treatment with CRT, and (4) successful completion of the treatment. Our complete patient selection criteria are shown in Figure 1. Overall, 781 patients with primary HNSCC were diagnosed or treated between January 1, 2005, and August 1, 2017. Of these, 460 (58.9%) met the selection criteria and had complete follow-up data.

### Data Collection, Treatment, and Follow-up

Demographic and clinical characteristics of study participants, including age, sex, social habits, comorbidities (respiratory, cardiovascular, immune, renal, endocrine), and previous cancer history, were collected by a detailed medical chart review. No genetic testing was feasible, as most patients had very limited biopsy tissue. Patients were dichotomized by NSAID use during treatment, which was considered positive (NSAID+) if it was noted in the electronic medical record that the patient was taking NSAIDs regularly (at least daily) at the time of consultation and during treatment. Clinical information, such as stage, grade, HPV status, anatomical subsite, treatment modality, cancer recurrence, survival status, survival duration, and cause of death were also collected. Local control (LC) was defined as the absence of disease within the initial treatment volumes. Distant failure was defined as progression at a site or an organ that is noncontiguous with the initial site of disease, typically the lungs in the setting of head and neck cancer. The cisplatin-based chemotherapy (weekly or every 3 weeks) and intensity-modulated radiation therapy regimens (70 Gy to the primary tumor and 56 Gy to the elective lymph nodes in 35 fractions) applied in this cohort have been previously described in detail.^13,14^ Patients could also be treated with induction chemotherapy, consisting of docetaxel, carboplatin, and fluorouracil, at the discretion of the medical oncologist.

### Statistical Analysis

Summary statistical analyses were performed from May 1, 2019, to March 17, 2020, and are provided for the demographic features of the patients included in this study. The associations of patient, tumor, and treatment characteristics with LC, DSS, and OS were examined using univariate analysis and multivariate Cox proportional hazard regression models. DSS is defined as death in a patient with a documented relapse, where patients are censored if there is death without known disease relapse. Analyses for the association between clinical outcomes and each particular variable were conducted. Only variables that were considered potential confounding factors were included before backward selection. All related factors that were significant at level α = .20 in the backward selection analyses were included in the final multivariate analysis. Survival estimates for OS and DSS were calculated using Kaplan-Meier survival curves. All tests were 2-sided, and *P* < .05 was considered statistically significant. SAS, version 9.4 (SAS Institute, Inc) was used for statistical analyses.

## Results

A total of 460 patients (median [interquartile range] age, 60 [53.9-65.6] years; 377 [82.0%] men and 83 [18.0%] women) were included in the analysis. At the time of consultation and during treatment, 201 (43.7%) patients were taking NSAIDs. This therapy consisted of low-dose 81-mg aspirin once daily for 109 of 201 (53.7%) patients, high-dose 325-mg aspirin once daily for 22 (10.9%) patients, and other NSAIDs (ibuprofen, naproxen, or meloxicam) for the remaining 70 (34.8%) patients. Median follow-up was 4.0 years (interquartile range, 2.5-6.4 years). Table 1 includes patient, tumor, and treatment characteristics. To improve readability, an additional row for number of patients missing data under each variable was not included in Table 1 but can be imputed. The majority of the characteristics were well balanced; however, there was a higher proportion of patients with type 1 and type 2 diabetes and coronary artery disease in the NSAID+ group and a small increase of patients with larynx and hypopharynx primary tumor sites in the group not taking NSAIDs. Additionally, a greater proportion of patients in the group not taking NSAIDs were current smokers. In the univariate analysis, current smokers vs former or never smokers had significantly worse LC (hazard ratio [HR], 2.30; 95% CI, 1.31-4.06; *P* = .004) and OS (HR, 1.67; 95% CI, 1.23-2.28; *P* = .001) and no significant change in DSS (HR, 1.62; 95% CI, 0.95-2.76; *P* = .08). In the multivariate analysis, current smokers vs former or never smokers had worse LC (HR, 5.70; 95% CI, 2.02-20.55; *P* = .03), DSS (HR, 3.97; 95% CI, 2.01-8.01; *P* = .03), and OS (HR, 3.94; 95% CI, 2.00-8.00; *P* < .001). Clinical tumor (T) stage was associated with worse LC in multivariate analysis (HR, 1.89; 95% CI, 1.42-2.52; *P* < .001) and worse OS in univariate (HR, 1.45; 95% CI, 1.27-1.67; *P* < .001) and multivariate (HR, 1.89; 95% CI, 1.42-2.52; *P* < .001) analysis. Clinical nodal (N) stage was associated with worse OS in multivariate analysis (HR, 4.44; 95% CI, 2.19-9.30; *P* < .001).

**Table 1.** Baseline Patient, Tumor, and Treatment Characteristics^a^

Variable	No. (%) of patients			*P* value
	Total cohort (N = 460)	No NSAID (n = 259)	NSAID+^b^ (n = 201)	
Age, median (SD) [range]	60 (9.5) [18-90]	58.5 (9.6) [33-90]	61.0 (9.3) [18-89]	.45
Sex				
Male	377 (82.0)	212 (81.9)	165 (82.1)	.98
Female	83 (18.0)	47 (18.1)	36 (17.9)	
Comorbidities				
Respiratory disease	79 (17.2)	44 (17.0)	35 (17.4)	.92
Diabetes mellitus	57 (12.4)	21 (8.1)	36 (17.9)	.002
Coronary artery disease	31 (6.7)	11 (4.2)	20 (10.0)	.02
History of stroke or VTE	21 (4.6)	10 (3.9)	11 (5.5)	.47
Alcohol consumption				
Never	70 (15.2)	36 (13.9)	34 (16.9)	.46
Former	98 (21.3)	52 (20.1)	46 (22.9)	
Current	276 (60.0)	160 (61.8)	116 (57.7)	
Not reported	16 (3.5)	11 (4.2)	5 (2.5)	
Smoking status				
Never	103 (22.4)	61 (23.6)	42 (20.9)	.55
Current	115 (25.0)	75 (29.0)	40 (19.9)	.03
Former	242 (52.6)	123 (47.5)	119 (59.2)	.14
T stage^c^				
T0	31 (6.8)	14 (5.5)	17 (8.5)	.37
T1	55 (12.1)	36 (14.1)	19 (9.6)	
T2	136 (29.9)	70 (27.3)	66 (33.2)	
T3	161 (35.4)	94 (36.7)	67 (33.7)	
T4a	63 (13.9)	36 (14.1)	27 (13.6)	
T4b	9 (2.0)	6 (2.3)	3 (1.5)	
N stage^c^				
N0	96 (30.0)	58 (22.6)	38 (18.9)	.71
N1	52 (11.4)	28 (10.9)	24 (11.9)	
N2a	43 (9.4)	19 (7.4)	24 (11.9)	
N2b	132 (28.8)	75 (29.2)	57 (28.4)	
N2c	85 (18.6)	49 (19.1)	36 (17.9)	
N3	50 (10.9)	28 (10.9)	22 (11.0)	
Overall clinical stage^c^				
0	1 (0.2)	0 (0.0)	1 (0.2)	.47
I	2 (0.4)	1 (0.2)	1 (0.2)	
II	23 (4.9)	12 (2.6)	11 (2.4)	
III	94 (20.7)	58 (12.8)	36 (7.9)	
IVA	287 (63.1)	156 (60.7)	131 (65.2)	
IVB	47 (10.3)	29 (11.3)	18 (4.0)	
IVC	1 (0.2)	1 (0.2)	0 (0)	
Primary tumor site				
Nasopharynx	15 (3.3)	8 (3.1)	7 (3.5)	.02
Oropharynx	242 (52.6)	125 (48.3)	117 (58.2)	
Oral cavity	20 (4.3)	11 (4.2)	9 (4.5)	
Larynx	112 (24.3)	72 (27.8)	40 (19.9)	
Hypopharynx	40 (8.7)	30 (11.6)	10 (5.0)	
Unknown primary	31 (6.7)	13 (5.0)	18 (9.0)	
HPV positive				
Yes	179 (38.9)	95 (36.7)	84 (41.8)	.44
No	94 (20.4)	52 (20.1)	42 (20.9)	
Unknown	187 (40.7)	112 (43.2)	75 (35.9)	
Treatment				
Chemoradiation + neck dissection	26 (5.7)	14 (5.4)	12 (6.0)	.47
ICT + chemoradiation	55 (12.0)	33 (12.7)	22 (10.9)	
Chemoradiation	379 (82.4)	212 (81.9)	167 (83.1)	

Abbreviations: HPV, human papillomavirus (p16 positive or HPV positive); ICT, induction chemotherapy; N, node; NSAID, nonsteroidal anti-inflammatory drug; T, tumor; VTE, venous thromboembolism.

^a^ To improve readability, an additional row for number of patients missing data under each variable was not included in Table 1 but can be imputed.

^b^ NSAID+ indicates that in the electronic medical record, the patient was noted to be taking NSAIDs regularly (at least daily) at the time of consultation and during treatment.

^c^ American Joint Committee on Cancer, 7th edition.

**Figure 2.**
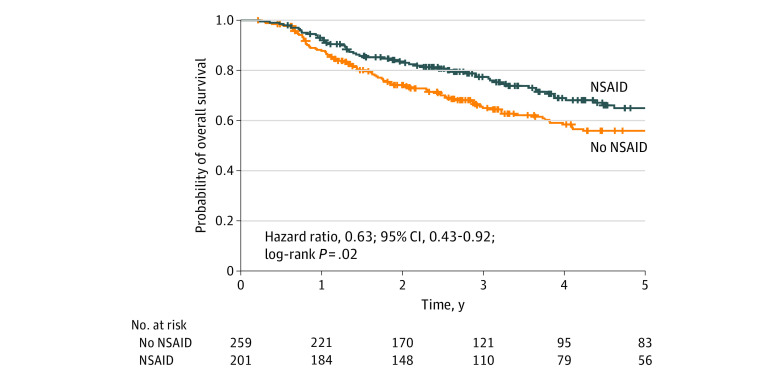
OS for Patients Taking NSAIDs vs Patients Not Taking NSAIDs NSAID indicates nonsteroidal anti-inflammatory drug.

**Figure 3.**
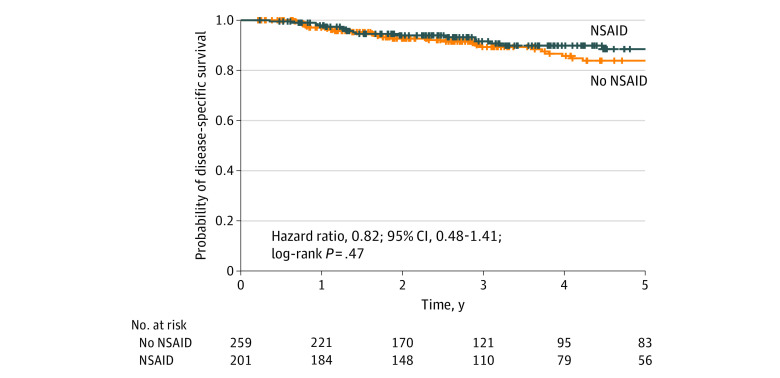
DSS for Patients Taking NSAIDs vs Patients Not Taking NSAIDs NSAID indicates nonsteroidal anti-inflammatory drug.

Figures 2 and 3 show the Kaplan-Meier curves for OS and DSS, respectively. Table 2 shows univariate and multivariate analyses of LC, DSS, and OS for NSAID use. In the univariate analysis, NSAID use was not associated with improved LC (HR, 0.59; 95% CI, 0.31-1.10; *P* = .10) or DSS (HR, 0.82; 95% CI, 0.48-1.41; *P* = .47) but was significant for OS (HR, 0.63; 95% CI, 0.43-0.92; *P* = .02). In the multivariate analysis, after adjusting for concurrent anticoagulant use and cardiac history, clinical T and N stage, disease subsite, and smoking status, NSAID use remained significantly associated with better OS (HR, 0.59; 95% CI, 0.38-0.90; *P* = .02). NSAID use was not associated with better DSS after multivariate analysis (HR, 0.98; 95% CI, 0.57-1.70; *P* = .44). NSAID use was not associated with better response to treatment (HR, 1.44; 95% CI, 0.91-2.27; *P* = .12) or distant failure (HR, 1.12; 95% CI, 0.68-1.84; *P* = .65). After excluding nasopharyngeal cancer and unknown primary tumor, NSAID use was associated with improved OS in univariate (HR, 0.67; 95% CI, 0.49-0.92; *P* = .01) and multivariate (HR, 0.62; 95% CI, 0.40-0.97; *P* = .04) analysis. Alcohol consumption was not associated with DSS (HR, 1.04; 95% CI, 0.73-1.48; *P* = .84) or OS (HR, 1.15; 95% CI, 0.87-1.52; *P* = .32). In the multivariate analysis, former smokers had worse DSS (HR, 2.67; 95% CI, 1.06-6.80; *P* = .04) and worse OS (HR, 3.80; 95% CI, 2.0-7.21; *P* < .001). Current smokers had no significant change in DSS (HR, 2.57; 95% CI, 0.91-7.24; *P* = .07) and significantly worse OS (HR, 4.02; 95% CI, 1.96-8.24; *P* < .001). In multivariate analysis, cardiac history was significantly associated with DSS (HR, 2.56; 95% CI, 1.31-5.05; *P* = .006), but there was no significant association with worse OS (HR, 1.55; 95% CI, 0.98-2.46; *P* = .06). NSAID use was not associated with distant failure (HR, 1.15; 95% CI, 0.70-1.89; *P* = .59). Survival curves demonstrated a significantly better OS at 5 years for patients taking NSAIDs compared with those who were not (63.6% [56 of 88 patients]; 95% CI, 58%-73% vs 56.1% [83 of 148 patients]; 95% CI, 50%-63%; *P* = .03).

**Table 2.** UVA and MVA of NSAID Use vs No NSAID Use

Variable	UVA		MVA	
	HR (95% CI)	*P* value	HR (95% CI)	*P* value
Local control	0.59 (0.31-1.10)	.10	0.60 (0.31-1.12)	.11
Disease-specific survival	0.82 (0.48-1.41)	.47	0.98 (0.57-1.70)	.44
Overall survival	0.63 (0.43-0.92)	.02	0.59 (0.38-0.90)	.02

Abbreviations: HR, hazard ratio; MVA, multivariate Cox proportional hazard regression model; NSAID, nonsteroidal anti-inflammatory drug; UVA, univariate Cox proportional hazard regression model.

## Discussion

This large, retrospective cohort study suggests a significant association with improved OS for patients with HNSCC taking NSAIDs during definitive CRT. Contrary to prior analyses, there was no evidence that concurrent NSAID use was associated with reduced distant failure.^15,16^ While the change in LC with NSAID use was not significant, future studies should continue to evaluate this possibility.

Few studies have previously examined the association between NSAID use during HNSCC treatment and patient survival. In a retrospective analysis examining 329 veterans, Lumley et al found that aspirin use after diagnosis and definitive treatment of HNSCC was associated with improved OS and DSS.^17^ Although our results suggest improved OS with NSAID use, we did not find it to be associated with improved DSS. A similar Korean study evaluated 1392 patients with HNSCC and found that postdiagnosis treatment with NSAIDs was not significantly associated with recurrence, survival, or second-cancer occurrence.^18^ This contrasts with our results, which could be related to the small sample size of patients taking NSAIDs or aspirin in their study (12.2%) compared with 43.7% in ours.

Hedberg et al reported specifically on patients with *PI3K*-altered HNSCC treated primarily with surgery and found a significant 5-year DSS and 5-year OS advantage for patients taking NSAIDs.^12^ Although we were unable to test for *PI3K*, our results suggest an OS advantage but not a DSS advantage. Hedberg et al suggest that *PIK3CA* variations may be a clinically useful marker to identify which patients with HNSCC will benefit from NSAID use^12^; however, until such testing is routine, the data presented here suggest that giving daily aspirin to patients with head and neck cancer who are receiving CRT may be associated with improved survival.

The exact mechanism by which NSAIDs are associated with survival in HNSCC remains unclear. Our study did not find a reduction in distant metastasis among patients taking NSAIDs, which is a potential explanation for the improved survival proposed by prior meta-analyses.^15,16^ There was also no DSS advantage for patients taking NSAIDs observed in our study. This suggests the observed survival advantage may be associated with the cardiovascular benefits of NSAIDs rather than any chemoprotective properties they may have, particularly because there was a higher proportion of patients with diabetes and coronary artery disease in the group taking NSAIDs. This is increasingly important because the risk of noncancer death now surpasses that of cancer death, with heart disease being the leading cause of noncancer mortality.^19^ The fact that anticoagulants were not associated with improved OS while NSAIDs were suggests that the cyclooxygenase mechanism may be a contributing factor to survival. This mechanism may be a combination of local recurrence reduction through cyclooxygenase inhibition and treatment of underlying cardiovascular disease.

### Limitations

This study is limited by several factors. Because of the nature of a retrospective review, the results are prone to information bias from miscoding of patient data and medication entry errors. In regard to NSAIDs, we had access to patient comorbidity data but did not have the reason why patients were prescribed regular NSAID use; nor did we have the duration of use. The vast majority of patients noted to be taking NSAIDs at the time of consultation were taking a “baby” (81-mg) aspirin, which was continued during CRT. Another limitation is the imbalance of primary tumor sites between the cohorts, with a small increase of patients with larynx and hypopharynx primary tumor sites and current smokers in the group not taking NSAIDs. This is adjusted for using multivariate analysis and does not alter the significance of the results. Despite these limitations, this review provides a large cohort of patients treated for HNSCC with CRT and concurrently taking NSAIDS.

## Conclusions

The results of this study suggest that there may be an OS advantage for patients taking NSAIDs during chemoradiation for HNSCC. Further studies examining this association are warranted.
